# Double Variational Binding—(SMILES) Conformational Analysis by Docking Mechanisms for Anti-HIV Pyrimidine Ligands

**DOI:** 10.3390/ijms160819553

**Published:** 2015-08-18

**Authors:** Mihai V. Putz, Nicoleta A. Dudaș, Adriana Isvoran

**Affiliations:** 1 Laboratory of Structural and Computational Physical-Chemistry for Nanosciences and QSAR, Biology-Chemistry Department, West University of Timisoara, Str. Pestalozzi No. 16, 300115 Timisoara, Romania; E-Mail: nicole_suceveanu@yahoo.com; 2 Environmental Advanced Researches Laboratories, Biology-Chemistry Department, West University of Timisoara, Str. Pestalozzi No. 16, 300115 Timisoara, Romania

**Keywords:** anti-HIV, 1,3-disubstituted uracil derivative, SMILES, ligand-receptor docking, chemical binding affinity, interacting amino acid

## Abstract

Variational quantitative binding–conformational analysis for a series of anti-HIV pyrimidine-based ligands is advanced at the individual molecular level. This was achieved by employing ligand-receptor docking algorithms for each molecule in the 1,3-disubstituted uracil derivative series that was studied. Such computational algorithms were employed for analyzing both genuine molecular cases and their simplified molecular input line entry system (SMILES) transformations, which were created via the controlled breaking of chemical bonds, so as to generate the longest SMILES molecular chain (LoSMoC) and Branching SMILES (BraS) conformations. The study identified the most active anti-HIV molecules, and analyzed their special and relevant bonding fragments (chemical alerts), and the recorded energetic and geometric docking results (*i.e.*, binding and affinity energies, and the surface area and volume of bonding, respectively). Clear computational evidence was also produced concerning the ligand-receptor pocket binding efficacies of the LoSMoc and BraS conformation types, thus confirming their earlier presence (as suggested by variational quantitative structure-activity relationship, variational-QSAR) as active intermediates for the molecule-to-cell transduction process.

## 1. Introduction

### 1.1. The General Anti-HIV Context

One of the preeminent challenges in molecular biology in the 21st century still remains in finding the weak spot of HIV’s infection. This is because the chemical binding mechanism of such fusion (producing a pore by which the virus discharged its capsid into cells’ nucleus, so infecting it forever) has not been elucidated [1].

The general mechanism of HIV’s invasive strategy is currently known, through the virus critical gp120 protein binding with the helper T cell’s receptors—the CD4 protein and its co-receptors CCR5/CXCR4. However, despite the virus crystal structure published back by 1998 [2] revealing the hydrophobic cavity at the gp120 center, the way the virus evades the immune system was a continuing open issue: the discovered pocket had some volume—thus allowing phenylalanine residue on CD4 but also for greater alkylating agents to fit into the cavity of gp120.

Then, the interaction between CD4 and gp120 was somehow blocked by the discovery of so-called NBD-556/557 [3], yet it was soon found it also enhanced the viral entry [4]. In order to prevent such adverse effects, the strategy was changed into mapping the amino acids, contributing to CD4-gp120 binding and to conformational changes when gp120 further binds with the T cell’s coreceptor, while selecting from the amino acids contributed to both phenomena than those involved in binding alone [5].

New inhibitors of HIV-1 viral entry were subsequently formulated by applying the molecular region/moiety strategy, by trial and error. Small molecules were derived from NBD-556 (e.g., guanidinium group mimicking the arginine residue on CD4): they do not elicit the conformational change in gp120, yet possessing some surprising mis-bindings on gp120 to the methionine residue, as revealed by the crystal structures of these compounds (called as JRC-II-191, (+)-DMJ-I-228/II-121) [6].

The passage to drug delivery was also made by further combining the DMJ compound with certain easily-elicited antibodies which, otherwise, are on their own ineffective to HIV infection, yet enhance their blocking feature this way complementing the vaccines [7].

Eventually, the antiretroviral prodrugs that bind directly to the virus, so preventing the binding with CD4 of T cells, were developed (the so-called BMS-663068 compound) and have already entered in the Phase IIb clinical trials [8].

Every year about 1.5–2 millions of people die from AIDS but a vaccine or a microbicide to prevent the infection is still yet to be found [9]. For the treatment of HIV infection, the targets for therapeutic intervention are one of the stages of the replicative cycle of HIV. In the last 30 years 26 compounds have been approved and classified according to the target they should inhibit: (i) Nucleoside reverse transcriptase inhibitors (NRTIs); (ii) Nucleotide reverse transcriptase inhibitors (NtRTIs); (iii) Non-nucleoside reverse transcriptase inhibitors (NNRTIs); (iv) Protease inhibitors (PIs); (v) Viral entry inhibitors (including coreceptor inhibitors [CRIs] and fusion inhibitors [FIs]); and (vi) Integrase inhibitors (INIs) [9,10,11].

### 1.2. The Anti-HIV Pyrimidine Derivatives

**Figure 1:**
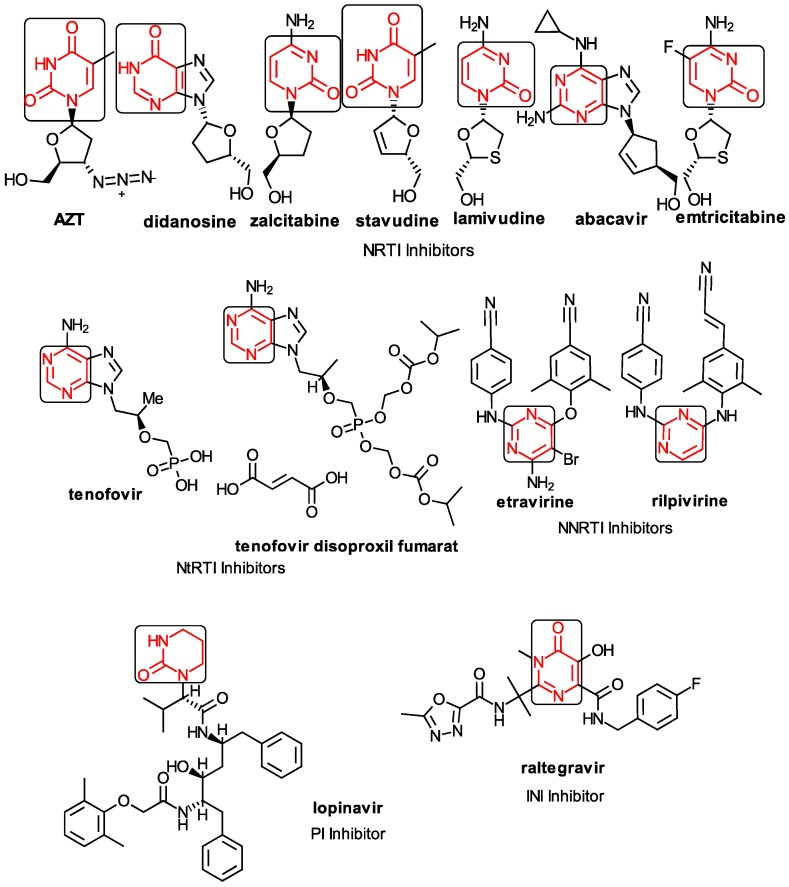
Pyrimidine derivatives used in the treatment of HIV/AIDS; the emphasized molecular fragments—also represented in the color red—are studied in this work.

Among the compounds approved by the FDA for clinical use, almost half of them are pyrimidine derivatives (see Figure 1) [9,10,11].

Pyrimidine derivatives occupy a leading position among the compounds investigated and/or approved in the treatment of HIV infection. AZT-zidovudine, the oldest drug used to treat HIV infection, was discovered serendipitously. It has its central core pyrimidine nucleus, and belongs to the class of NRTI inhibitors [9,10]. Most pyrimidine derivatives are from the NRTI class, namely [12,13,14,15,16,17,18,19] AZT, didanosine-ddI, zalcitabine-ddC, stavudine-d4T, lamivudine-3TC, abacavir-ABC, and emtricitabine-(−)-FTC. Similarly, pyrimidine derivatives in other classes include:

➢ The NtRTI is a pyrimidine derivative investigated in microbicide preparations, e.g., tenofovir (TFV) and specifically its precursor—the tenofovir disoproxil fumarate (TDF, PMPA) [12,13,14,15,16,17,18,19];
➢ The second-generation of NNRTI inhibitors: etravirine (ETR, TMC125) and rilpivirine (RPV, TMC278) [12,13,14,15,16,17,18,19,20,21,22];
➢ The PI inhibitor: lopinavir (ABT-378) [12,13,14,15,18,19,23];
➢ The INI inhibitor: raltegravir (RAL, MK-0518) [12,13,14,15,17,18,19,24] (see Figure 1) [9,10,11,12,13,14,25,26,27,28,29].

New compounds are designed from the need for formulating derivatives with better anti-HIV activity that are active against various mutations of the virus with high selectivity, no side effects, and reduced toxicity.

Some pyrimidine derivatives are in different stages of clinical testing.

Dapivirine (TMC 120) was initially tested as a NNRTI inhibitor (close analogs etravirine and rilpivirine having been so approved), but is now in clinical trials as a microbicide in combination with TFV. Although emivirine (MKC-442, I-EBU) has passed all testing phases, it has still not been approved for marketing.

**Figure 2:**
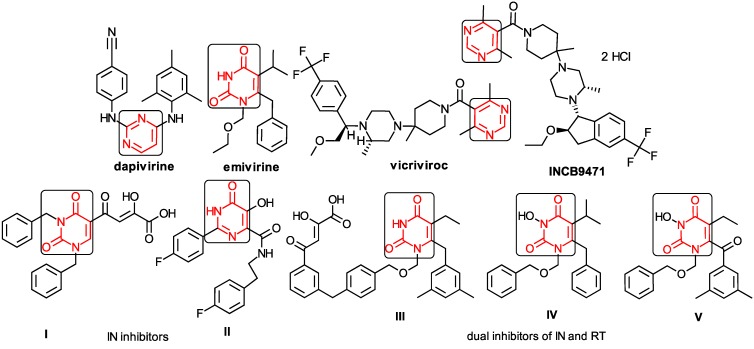
Pyrimidine derivatives which have been tested or have reached in different stages of clinical trials for treatment of HIV/AIDS; the emphasized molecular fragments—also represented in the color red—are studied in this work.

The CRI inhibitor (SCH-D, SCH-417690) behaves like a CCR5 antagonist but another CRI inhibitor INCB-9471 acts like an uncompetitive allosteric antagonist of CCR5 (Figure 2) [10,11,12,13,14,15,16,25,26,27,28,29].

Many INI inhibitors are in various stages of research. Examples of some pyrimidine-diketoacids (PY-DKA) are:

➢ The 4-(1,3-dibenzyl-1,2,3,4-tetrahydro-2,4-dioxopyrimidin-5-yl)-2-hydroxy-4-oxo-but-2-enoic acid. It corresponds to compound I in Figure 2 being a diketo acid bearing a nucleobase scaffold, which opened a new research direction in the chemistry of INI derivatives) [17,18];
➢ The 5-hydroxy-6-oxo-1,6-dihydropyrimidine-4-carboxamide analog: *N*-(4-fluorobenzyl)-2-(4-fluorophenyl)-5-hydroxy-6-oxo-1,6-dihydropyrimidine-4-carboxamide corresponding with compound II in Figure 2 [19].

On the other side, among the RT and IN dual inhibitors one may list compound III in Figure 2, the (*Z*)-4-(3-(4-(((6-(3,5-dimethylbenzyl)-5-ethyl-2,4-dioxo-3,4-dihydropyrimidin-1(2H)-yl)methoxy)methyl)benzyl) phenyl)-2-hydroxy-4-oxobut-2-enoic acid, which was obtained from rational design of RT/IN dual inhibitors based on HEPT NNRTI and DKA IN inhibitors [20], and the N-3 hydroxylated pyrimidine-2,4-diones illustrated as compounds IV and V in the Figure 2, respectively [21,22].

### 1.3. The Anti-HIV Mechanisms of Action

The most attractive target in anti-HIV chemotherapy is reverse transcriptase (RT) because of its key role in virus replication. The HIV-1 RT is a asymmetric heterodimer with enzymatic activity, made out of two subunits: p66 subunit with 560 amino acids and p51 subunit with 440 amino acids [12,13,23,24,30,31].

The p51 subunit contains the same subdomains as the p66 subunit but adapts a different spatial arrangement from the p66 subunit and has no catalytic function. The p51 subunit plays only a role in maintaining the entire structure of the RT [12,13,23,24,30,31,32,33].

HIV-RT structure looks like a right hand, with the subunit p66 containing both the polymerase and RNase H (ribonuclease family H) active sites. The residues 1–85 form the fingers, residues 86–117 and 156–237 form the palm and residues 238–318 occupy the thumb regions. The connection region contains residues 319–426 and the RNase H subdomains host the residues 427–560 [12,13,23,24,30,31,32,33]. Finger and thumb domains are little flexible. However, palm and thumb domains form a nucleic acid binding cleft where the active site of HIV-RT is located [12,13,23,24,30,31,32]. The palm subdomain of RT contains the polymerase active site by the catalytic triad Asp110, Asp185 and Asp186. The NNRTI-binding pocket (NNBP) is located in p66 subunit, at 10 Å away from the catalytic site of the palm subdomain.

Nevertheless, the NNRTI-binding pocket exists only when an inhibitor is bound to the enzyme and consists of aromatic and aliphatic hydrophobic residues such as Tyr181, Tyr188, Phe227, Trp 229, Tyr232 and Pro59, Leu100, Val106, Val179, Leu234, and Pro236. It is also comprised of five hydrophilic residues namely Lys101, Lys103, Ser105, Asp132, and Glu224. The additional residues that belong to the p51 subunit are Ile135, Glu1138, and Thr139 [23,24,31,32,33,34,35,36].

The NNBP has been described to feature three channels designated as the entrance, groove, and tunnel [37,38,39,40]:

➢ The protein/solvent interface is close to Pro236, Val106 and Leu234;
➢ The largely open region in front of Lys101, Lys103, Glu138, and Val179 is considered to form the entrance channel for the NNRTI binding site;
➢ The tunnel is lined by Tyr181, Tyr188, Trp229, and Phe227, which leads towards the polymerase active site;
➢ The groove is lined by Phe227, Tyr318, Pro225, Pro236.

The polymerase activity of the HIV RT has similar features to most DNA polymerases with a higher affinity to RNA as a template, while the RNase H activity catalyze the degradation of the template RNA in the RNA/DNA hybrid during the reverse transcription [12].

The NNRTI and N(t)RTI inhibitors are targeting the reverse transcriptase (RT). N(t)RTI are substrate competitive inhibitors, which are analogues of natural deoxynucleotide required for viral DNA synthesis [10,11,13,25,26,27]. In order to be activated, they must be phosphorylated to the triphosphate form and, because they lack the 3ʹ-hydroxyl on desoxyribose, they do not allow the incorporation of the next deoxynucleotide, thus stopping the synthesis of viral DNA [10,11,13,25,26,27]. Yet, the big advantage is that they are active against both HIV-1 and HIV-2 [10,11,13,25,26,27].

The NNRTIs are uncompetitive inhibitors that act by allosteric inhibition of DNA polymerization. They inhibit RT by binding to a deep hydrophobic pocket different from the NRTI and not far from the active site of the p66 subunit, *i.e.*, in the palm subdomain adjacent to the base of the thumb subdomain, referred to as the NNRTI binding pocket–NNIBP [10,11,12,13,23,24,25,26,27,30,31]. During the binding of NNRTI a local conformational change of opening the NNIBP occurs and, after the binding, it changes its conformation again, closing the pocket [24,30,31,41]. The NNBP presents different conformations depending on the type of compound NNRTI bound [12,24,31,34]. The first generation of NNRTI derivatives have more rigid structures, shapes similar to a butterfly, with a hydrophilic body and wings formed of hydrophobic groups. The second generation of NNRTI inhibitors have more flexible structures, shapes similar to a horseshoe, with polar center and hydrophobic side wings (e.g., ripivirine) [24,31,34]. The NNRTIs are highly specific, causing fewer side effects but they cannot be used in the treatment of HIV-2 since the resistance to them develops rather quickly by moving the entry in NNIBP, therefore casing the loss/change of the hydrophobic interactions and of steric hindrance [23,24,30,31,34,35,36].

When NNRTIs bind to their pocket (NNBP) in the HIV-1 RT some conformations are changing with structural-functional consequences [10,12,13,23,24,30,31,32,33,42,43,44]:

➢ The restriction of thumb mobility;
➢ Distortion of the catalytic triad;
➢ Repositioning of the primer grip;
➢ and loosening the thumb and fingers clamp.

These changes will lead to the formation of the NNBP whose volume is more than twice than that occupied by most of NNRTIs (620–720 Å^3^) [10,12,13,23,24,30,31,32,33,42,43,44]. The Tyr181 and Tyr188, despite originally pointing to the hydrophobic center of the pocket, will rotate towards the direction of the catalytic site. Due to this rotation, the catalytic residues will shift their position around 2 Å; therefore, the entry in the pocket is flanked by Pro225 and Pro236 which are located on flexible chains. At the interface between p66 (surrounded by Leu100, Lys101, Lys103 Val179 and Tyr181) and p51 (surrounded by Glu1138 and Thr1139) a solvent accessible area is formed [10,12,13,23,24,30,31]. The flexibility is crucial for allowing opening and closing “the mouth” for the entrance of the NNRTI [12]. The overall shape of the pocket does not vary significantly even with chemically very different NNRTI derivatives [23]. In addition to the hinge movement of the thumb, the p66 connection and RNase H subdomains are also distorted from the normal position in an unbound enzyme [12]. Although NNRTIs bind to RT around 60 Å away from the RNase H active site, several studies have demonstrated that they can either partially inhibit or accelerate RNaseH activity [23,45,46].

### 1.4. The NNRTIs–RT Basic Interactions

Some interactions are crucial and play an important role in binding NNRTIs to RT [10,12,13,23,24,30,31].

Very important are the hydrophobic π–π interactions present between π-electron containing components of the inhibitors and the aromatic residues in RT (Tyr181, Tyr188, Trp229) [10,12,13,23,24,30,31]. Equally important are the van de Waals interactions of the inhibitor with various positions in RT (Leu100, Val106, Val179, Leu234) which may increase their affinity to the enzyme, while interactions with Lys101, Lys103 and Glu1138 ensure the possibility of catching electrostatic interactions. Likewise, the H-bonds between NNRTIs and the enzyme can be an important anchor for them [10,12,13,23,24,30,31].

Loss of some key interactions will significantly reduce the potency of the inhibitor.

Many NNRTIs are active against HIV-1 RT and inactive to HIV-2 RT. This fact can be explained by structural differences between the RT HIV-1 and HIV-2. Namely, the HIV-1 RT has a tyrosine residue in the 181 position that plays an important role in interacting with many NNRTIs, while HIV-2 RT has an isoleucine residue in the 181 position [10,12,13,23,24,30,31,47].

Among the most important interactions that an NNRTI should retain with RT while it wiggles and jiggles in NNBP are [24]:

➢ Hydrophobic sandwiches;
➢ A characteristic hydrogen bond with the Lys101 main-chain carbonyl;
➢ And water-mediated hydrogen bonds.

Yet, a series of 1-substituted-3-(3,5-dimethylbenzyl) uracils do not form this H-bond as was shown by Maruyama, *et al.*, while the compounds still retain affinity with RT indicating that the H-bond is not essential for docking of 1-substituted-3-(3,5-dimethylbenzyl) uracils [48].

The biggest challenge for researchers is to identify new compounds that do not lead to resistance and also which must be active against various mutations in the virus that already exist.

The NNRTI resistance mutations impact the binding of the molecules in the NNBP, but some of those mutations have also been described to influence functionalities of RT other than DNA polymerization (e.g., the V106A and P236L mutations cause a slowing RNaseH cleavage activities while the Y181C mutation shows an acceleration of RNaseH cleavage activities) [13,23,49]. Over 40 amino acid substitutions (all located in the NNRTI binding pocket) have been identified to be associated with NNRTI resistance. They are mainly present in domains which include amino acids 98–108, 178–190, and 225–238 of the p66 subunit (the most usual mutations observed in HIV-1 RT relevant to clinical NNRTI-resistance are K103N, Y181C, Y318F, Y188C, and L100I) [12,13,23,24,30].

The mutation of an aromatic tyrosine to a non-aromatic cysteine causes a dramatic change from a hydrophobic environment to a hydrophilic environment of the binding pocket; therefore, most of the possible hydrophobic contacts are abolished [50]. Mutations in the p51 subunit at the Glu138 position causes resistance to NNRTIs, as well as mutations N384I, T369I, and E399D have been shown to confer resistance to both NRTIs and NNRTIs [12,13,23,24,30,51,52].

### 1.5. Overview of the Present Study

In this broad context, the present paper contributes for the HIV’s “weak spot” [1] by small molecules, the pyrimidines in this case. Accordingly, the paper will unfold the follows:

➢ Section 2 presents the working pyrimidine series, their structural roots, as well as their SMILES (simplified molecular input line entry system) conformations, which were created via the controlled breaking of chemical bonds;
➢ The generated longest SMILES molecular chain (LoSMoC) and Branching SMILES (BraS) cases are further considered in Section 3 as the variational transformation into anti-HIV docking action;
➢ The Section 4 interprets the computational docking results in variational Genuine-LoSMoC-Branching (BraS) form while selecting the most versatile pyrimidine molecule able to change its conformation (variationally).

The current advanced double-variational algorithm conceptually-computationally adapts to the HIV pocket variational shielding—*i.e.*, by combining both binding and conformation information towards the docking of the most active anti-HIV ligand. Reference to available knowledge in the anti-HIV by HEPTs research field and especially related with the amino acid residues in bonding is systematically pursued. The paper closes with general lessons and conclusions on combining the binding with conformational change of small molecules aiming at anti-HIV activity.

## 2. SMILES of Anti-HIV Pyrimidines

### 2.1. Presenting the Anti-HIV HEPT Derivatives

Post 1996, the most effective treatment for AIDS is the highly-active antiretroviral therapy (HAART). To this end the NNRTIs are important components of HAART with high antiviral potency, high specificity, and low cytotoxicity [10,23,53,54,55].

The NNRTI class is characterized by a high chemical diversity, with more than 50 families of molecules that have been reported so far [10,23,53,54,55].

**Figure 3:**
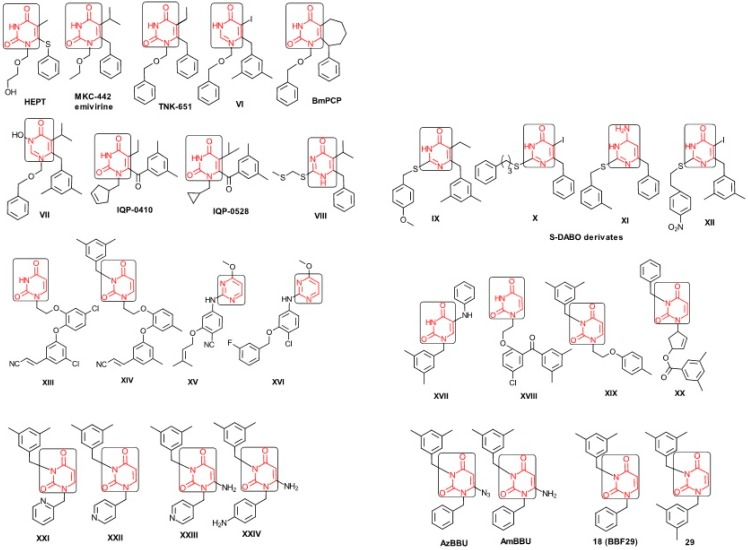
Pyrimidine derivatives already tested as NNRTI; the emphasized molecular fragments—also represented in the color red—are studied in this work. See text for details.

The era of the pyrimidine NNRTIs started with the discovery of the anti-HIV-1 activity of the HEPT derivatives (1-[(2-hydroxyethoxy)methyl]-6-(phenylthio)thymine) [10,23,56,57] (Figure 3).

Concurrently, the TIBO derivatives (tetrahydroimidazo[4,5,1-jk][1,4]-benzodiazepine-2(1H)-one and –thione) were discovered as specific HIV-1 inhibitors targeting the HIV-1 RT, nevirapine, delavirdine, and efavirenz being also approved and commercialized for clinical use [10,23,58] (Figure 3).

The MKC-442 (emivirine) has HEPT origins (Figure 3), even if it was very promising, it was abandoned after phase III of clinical trials [10,14,15,28].

In addition to HEPT origins is TNK-651 (Figure 3), which has shown to have great potential as a NNRTI. For it, virus mutations and rapid installation of resistance were observed, along the decrease of PI bioavailability that was tested (as happened in the case of emvirine). As a consequence, any further development of this compound was not considered [10,15,21,59].

The HEPT-like compounds TNK-651 and emvirine were a source of inspiration for future compounds such as those of Figure 3, e.g., [10,12,13,15,21,59,60]:

➢ The compound VI (1-benzyloxymethyl-6-(3,5-dimethylbenzyl)-5-iodouracil), has potent NNRTI activity against HIV-1 strains resistance (through a halogen at the C-5 position and meta-substituents on the C-6 aromatic moiety) [60];
➢ The compound BmPCP (1-[(benzyloxy)methyl]-9-phenyl-6,7,8,9-tetrahydro-1H-cyclohepta[d]pyrimidine-2,4-(3H,5H)-dione);
➢ The compound VII (6-benzyl-1-(benzyloxymethyl)-3-hydroxy-5-isopropyl-uracil) which proved to be a potential dual inhibitor behaving both as NNRTI and INI alike;
➢ Worth mentioning the pyrimidin-diones (PYD) among potential dual inhibitors, this time as RT and Viral entry inhibitors, as we may list IQP-0410 (SJ-3366) and IQP-0528 compounds featuring better inhibitory activity than any congener HEPT derivatives [61,62,63,64,65,66].

They all have shown very good inhibitory activity *versus* many strains and subtypes of HIV being very potent NNRTIs against HIV-1. While they are not inhibiting HIV RT-2, they still inhibit the entry step for both HIV-1 and HIV-2 [61,62,63,64,65,66]. Moreover, these compounds act synergistically in combination with other antiretrovirals (ARV) with no observed toxicity antagonistic effects. For instance, from Figure 3, the IQP-0410 proved to be the best candidate for oral therapy in a combination with another ARV, such that the resultant combination targeting at least threefold in HIV inhibition; also IQP-0528 was developed in the treatment of prevention, as a local microbicide in different forms and combinations [61,62,63,64,65,66].

On the other side, the TIBO synthesis path successively led to DAPY–diaril-pyrimidine etravirine (TMC278) and rilpivirine (TMC120), and dapivirine (TMC125) of Figure 2 and Figure 3, which have so far been approved for clinical use [10,12,13,23,67]. Since DAPY NNRTI inhibitors were first produced, many derivatives with modifications on the structural diversity of the linker between the right benzene ring and the central pyrimidine ring have been developed. The left wing of the DAPY structure was confirmed as the indispensable pharmacophore, e.g., the related compounds CH-DAPY, CH(OH)-DAPY, CR(OH)-DAPY, CH(CN)-DAPY, C(=NOH)-DAPY, O-DAPY, pDAPY–piperidinylamino-diarylpyrimidine, and CAPY–cycloalkyl arylpyrimidines [37,50,67,68,69,70,71,72,73,74].

Another family intensively researched are the DABO derivatives (dihydro-alkoxy-benzyl-oxopyrimidine), a molecular class having in the C-2 position the presence of an alkoxy group for DABO derivatives. Accordingly, the compounds like alkylthio for S-DABO or as alkylamino for NH-DABO are important for their activity as NNRTI. This is because the C-2, C-5, and C-6 substituent effects were tightly linked: the optimal moieties at positions 5 and 6 of the pyrimidine nucleus are dependent on the nature of the C-2 side chain. Some representatives from S-DABO family are compounds VIII–XII in Figure 3, while some DABO derivatives are currently tested as microbicides or were obtained hybrids DAPY-DABO with a very good inhibitory activity [75,76,77,78,79,80,81,82,83].

The literature also communicates very different structures of NNRTI containing the pyrimidine core and exhibiting very good anti-HIV activity, as seen from Figure 3, the pyrimidine-catechol-diether compounds XIII and XIV, or compound XV and compound XVI (representative for a novel class of 2-pyrimidinylphenylamine derivatives) [39,40,84,85,86]. Similar compounds are 1,3-dibenzyl-uracil derivates, e.g., benzylated pyrimidines, being substituted in N1 and/or N3 position of pyrimidine nucleus by Maruyama *et al.* [28,48,87,88,89,90,91]; they have been synthesized and tested as the compounds XVII and XX in Figure 3 (see Novikov *et al.* [92,93,94,95]).

The HEPT derivatives inspired Maruyama, *et al.* to identify for 1,3-disubstituted uracil derivatives wwith very good anti-HIV-1 activity as NNRTIs, and in Figure 3 presented as the compounds 18 (BBF29) and 29—representative for the series of our study—see also Table 1 and forthcoming discussion. Based on compound 18 other compounds with increased anti-HIV-1 activity have been developed, namely (cf. Figure 3) [28,48,87,88,89,90,91]:

➢ The compounds XXI (3-(3,5-dimethylbenzyl)-1-(2-pyridinylmethyl)-2,4(1H,3H)-pyrimidinedione) and XXII (3-(3,5-dimethylbenzyl)-1-(4-pyridinylmethyl)-2,4(1H,3H)-pyrimidinedione);
➢ The foremost representative compounds AzBBU (6-azido-1-benzyl-3-(3,5-dimethylbenzyl) uracil) presumed to be reduced by metabolic pathway in AmBBU (6-amino-1-benzyl-3-(3,5-dimethylbenzyl)uracil;
➢ The derivates of AmBBU as compounds XXIII (6-amino-3-(3,5-dimethylbenzyl)-1-(4-pyridinylmethyl)-uracil and XXIV (6-Amino-3-(3,5-dimethylbenzyl)-1-(4-aminobenzyl)-uracil.

Nevertheless, dedicated studies confirm that one of the most usual mutations (Y181C) seems to be sufficient for the acquisition of resistance to AzBBU and AmBBU, while other common NNRTI resistance mutations, such as K101E, K103N, and Y188C, were not identified for which the compound 18 shares similar properties [28].

### 2.2. SMILES Forms for Working HEPT Derivatives

From this point onward, one employs the working molecules under those actually most likely to form, targeting the considered end-point, namely the anti-HIV activity, as produced by uracil-based pyrimidines [87,96]. They are represented by the (gas-phase) genuine molecular structure alongside the two forms of their simplified molecular input line entry system (SMILES) structure, as presented in Table 1, respectively [9,96,97,98,99].

**Table 1:** Working molecules (IUPAC name and molecular weight MW are indicated ) and their corresponding SMILES topology, *i.e.*, the longest SMILES molecular chain (LoSMoC) as upper entry and the Branching SMILES (BraS) as down entry, for each pyrimidine structure considered, manifesting antiviral activity of 1,3-disubstituted uracils against human immunodeficiency virus (HIV-1) [87], with AIDS code indicated [96], respectively. SMILES legend is: 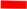 principal SMILES chain; 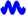 secondary SMILES branch; 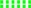 tertiary SMILES branch; 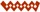 **quaternary** SMILES branch; = double bond; # triple bond; /,\ directional bonds; ( ) branch; C, N, F, S, I–atoms present in the molecule; c, n–atoms place in an aromatic ring; C_1/2/3_, N_1/2_, c_1/2/3_, n_2_–connectivity points [97,98,99].

No.	Structure 2D	IUPAC Name	*M*_W_	AIDS Code	SMILES Configurations	
					LoSMoC	Code LoSMoC
					BraS	Code BraS
**1**	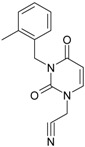	[3-(2-Methyl-benzyl)-2,4-dioxo-3,4-dihydro-2H-pyrimidin-1-yl]-acetonitrile	255.28	AIDS352092	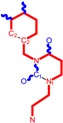	N#CCN1/C=C\C(=O) N(C1=O)Cc2ccc(C)c(C)c2
					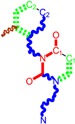	O=C1N(Cc(c(C)cc2)cc2) C(N(/C=C1\)CC#N)=O
**2**	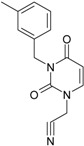	[3-(3-Methyl-benzyl)-2,4-dioxo-3,4-dihydro-2H-pyrimidin-1-yl]-acetonitrile	255.28	AIDS352093	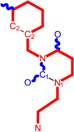	N#CCN1/C=C\C(=O) N(C1=O)Cc2cccc(C)c2
					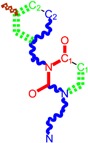	O=C1N(Cc(cc(C)c2)cc2) C(N(/C=C1\)CC#N)=O
**3**	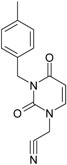	[3-(4-Methyl-benzyl)-2,4-dioxo-3,4-dihydro-2H-pyrimidin-1-yl]-acetonitrile	255.28	AIDS352094	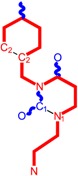	N#CCN1/C=C\C(=O) N(C1=O)Cc2ccc(C)cc2
					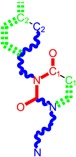	O=C1N(Cc(ccc2C)cc2) C(N(/C=C1\)CC#N)=O
**4**	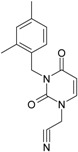	[3-(2,4-Dimethyl-benzyl)-2,4-dioxo-3,4-dihydro-2H-pyrimidin-1-yl]-acetonitrile	269.30	AIDS352888	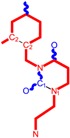	N#CCN1/C=C\C(=O) N(C1=O)Cc2ccc(C)cc2C
					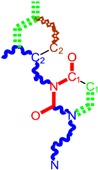	O=C1N(Cc2c(cc(cc2)C)C) C(N(/C=C1\)CC#N)=O
**5**	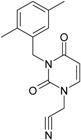	[3-(2,5-Dimethyl-benzyl)-2,4-dioxo-3,4-dihydro-2H-pyrimidin-1-yl]-acetonitrile	269.30	AIDS352889	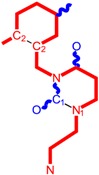	N#CCN1/C=C\C(=O) N(C1=O)Cc2cc(C)ccc2C
					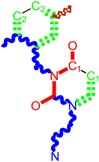	O=C1N(Cc(cc(C)c2)c(c2)C) C(N(/C=C1\)CC#N)=O
**6**	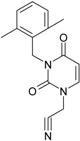	[3-(2,6-Dimethyl-benzyl)-2,4-dioxo-3,4-dihydro-2H-pyrimidin-1-yl]-acetonitrile	269.30	AIDS352890	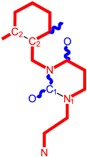	N#CCN1/C=C\C(=O) N(C1=O)Cc2c(C)cccc2C
					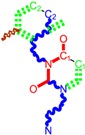	O=C1N(Cc(c(C)cc2)c(C)c2) C(N(/C=C1\)CC#N)=O
**7**	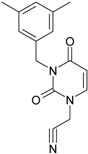	[3-(3,5-Dimethyl-benzyl)-2,4-dioxo-3,4-dihydro-2H-pyrimidin-1-yl]-acetonitrile	269.30	AIDS352095	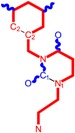	N#CCN1/C=C\C(=O) N(C1=O)Cc2cc(C)cc(C)c2
					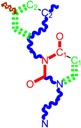	O=C1N(Cc(cc(C)c2)cc2C) C(N(/C=C1\)CC#N)=O
**8**	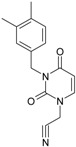	[3-(3,4-Dimethyl-benzyl)-2,4-dioxo-3,4-dihydro-2H-pyrimidin-1-yl]-acetonitrile	269.30	AIDS352891	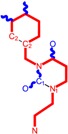	N#CCN1/C=C\C(=O) N(C1=O)Cc2ccc(C)c(C)c2
					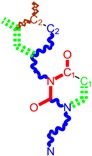	O=C1N(Cc(cc(c2C)C)cc2) C(N(/C=C1\)CC#N)=O
**9**	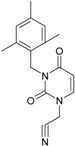	[3-(2,4,6-Trimethyl-benzyl)- 2,4-dioxo-3,4-dihydro-2H-pyrimidin-1-yl]-acetonitrile	283.33	AIDS352892	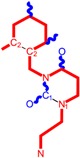	N#CCN1/C=C\C(=O) N(C1=O)Cc2c(C)cc(C)cc2C
					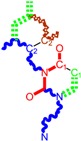	O=C1N(Cc2c(cc(cc2C)C)C) C(N(/C=C1\)CC#N)=O
**10**	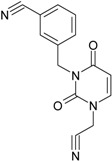	[3-(3-Cyanophenyl)methyl-2,4-dioxo-3,4-dihydro-2H-pyrimidin-1-yl]-acetonitrile	266.26	AIDS352893	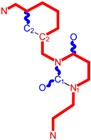	N#CCN1/C=C\C(=O) N(C1=O)Cc2cccc(c2)C#N
					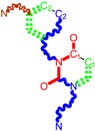	O=C1N(Cc(cc(C#N)c2)cc2) C(N(/C=C1\)CC#N)=O
**11**	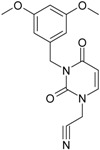	[3-(3,5-Dimethoxy-benzyl)-2,4-dioxo-3,4-dihydro-2H-pyrimidin-1-yl]-acetonitrile	301.30	AIDS352897	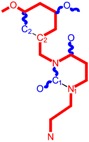	N#CCN1/C=C\C(=O)N(C1=O) Cc2cc(OC)cc(c2)OC
					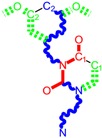	O=C1N(Cc(cc2OC)cc(OC)c2) C(N(/C=C1\)CC#N)=O
**12**	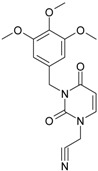	[3-(3,4,5-trimethoxy-benzyl)-2,4-dioxo-3,4-dihydro-2H-pyrimidin-1-yl]-acetonitrile	331.33	AIDS352898	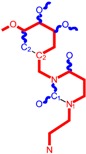	N#CCN1/C=C\C(=O)N(C1=O) Cc2cc(OC)c(OC)c(c2)OC
					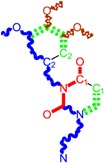	O=C1N(Cc2cc(c(OC)c(OC)c2)OC) C(N(/C=C1\)CC#N)=O
**13**	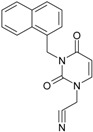	(3-Naphthalen-1-ylmethyl-2,4-dioxo-3,4-dihydro-2H-pyrimidin-1-yl)-acetonitrile	291.31	AIDS352899	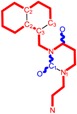	N#CCN1/C=C\C(=O)N (C1=O)Cc3c2ccccc2ccc3
					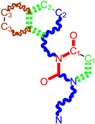	O=C1N(Cc(c(cc3)c(cc3)c2)cc2) C(N(/C=C1\)CC#N)=O
**14**	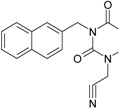	(3-Naphthalen-2-ylmethyl-2,4-dioxo-3,4-dihydro-2H-pyrimidin-1-yl)-acetonitrile	291.31	AIDS352900	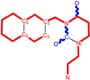	N#CCN1/C=C\C(=O)N (C1=O)Cc3cc2ccccc2cc3
					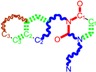	O=C1N(Cc(cc(ccc3)c2c3)cc2) C(N(/C=C1\)CC#N)=O
**15**	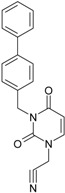	(3-Biphenyl-4-ylmethyl-2,4-dioxo-3,4-dihydro-2H-pyrimidin-1-yl)-acetonitrile	317.35	AIDS352901	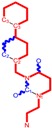	N#CCN1/C=C\C(=O)N (C1=O)Cc2ccc(cc2)c3ccccc3
					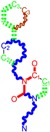	O=C1N(Cc(c2)ccc(c(cc3)ccc3)c2) C(N(/C=C1\)CC#N)=O
**16**	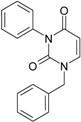	1-Benzyl-3-phenyl-1H-pyrimidine-2,4-dione	278.31	AIDS352902	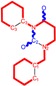	c1ccccc1CN2/C=C\C(=O) N(C2=O)c3ccccc3
					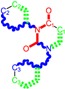	O=C1N(c(cc2)ccc2)C(N(/C=C1\) Cc(ccc3)cc3)=O
**17**	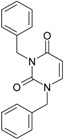	1,3-Dibenzyl-1H-pyrimidine-2,4-dione	292.34	AIDS352903	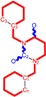	c1ccccc1CN2/C=C\C(=O) N(C2=O)Cc3ccccc3
					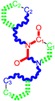	O=C1N(Cc(ccc2)cc2)C (N(/C=C1\)Cc(ccc3)cc3)=O
**18**	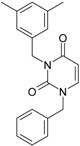	1-Benzyl-3-(3,5-dimethyl-benzyl)-1H-pyrimidine-2,4-dione	320.39	AIDS352096	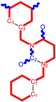	c1ccccc1CN2/C=C\C(=O) N(C2=O)Cc3cc(C)cc(C)c3
					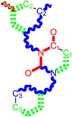	O=C1N(Cc(cc(C)c2)cc2C)C (N(/C=C1\)Cc(ccc3)cc3)=O
**19**	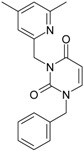	1-Benzyl-3-(4,6-dimethyl-pyridin-2-ylmethyl)-1H-pyrimidine-2,4-dione	321.38	AIDS352904	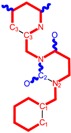	c1ccccc1CN2/C=C\C(=O) N(C2=O)Cc3nc(C)cc(C)c3
					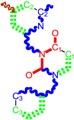	O=C1N(Cc(cc(C)c2)nc2C)C (N(/C=C1\)Cc(ccc3)cc3)=O
**20**	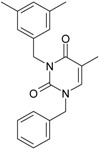	1-Benzyl-3-(3,5-dimethyl-benzyl)-5-methyl-1H-pyrimidine-2,4-dione	334.42	AIDS352905	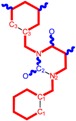	c1ccccc1CN2/C=C\(C)C(=O) N(C2=O)Cc3cc(C)cc(C)c3
					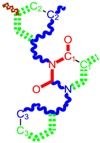	c1ccccc1CN2/C=C\(C)C(=O) N(C2=O)Cc3cc(C)cc(C)c3
**21**	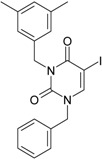	1-Benzyl-3-(3,5-dimethyl-benzyl)-5-iodo-1H-pyrimidine-2,4-dione	446.29	AIDS352906	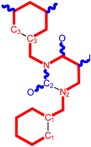	c1ccccc1CN2/C=C\(I)C(=O) N(C2=O)Cc3cc(C)cc(C)c3
					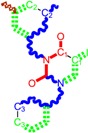	O=C1N(Cc(cc(C)c2)cc2C) C(N(/C=C1\I)Cc(ccc3)cc3)=O
**22**	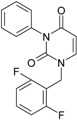	1-(2,6-Difluoro-benzyl)-3-phenyl-1H-pyrimidine-2,4-dione	314.29	AIDS352907	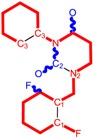	Fc1cccc(F)c1CN2/C=C\C (=O)N(C2=O)c3ccccc3
					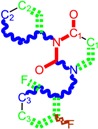	O=C1N(c(cc2)ccc2)C(N(/C=C1\) Cc(c(F)cc3)c(F)c3)=O
**23**	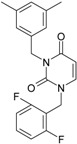	1-(2,6-Difluoro-benzyl)-3-(3,5-dimethyl-benzyl)-1H-pyrimidine-2,4-dione	356.37	AIDS352908	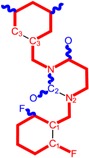	Fc1cccc(F)c1CN2/C=C\C(=O) N(C2=O)Cc3cc(C)cc(C)c3
					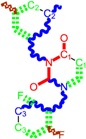	O=C1N(Cc(cc(C)c2)cc2C)C (N(/C=C1\)Cc(c(F)cc3)c(F)c3)=O
**24**	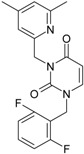	1-(2,6-Difluoro-benzyl)-3-(4,6-dimethyl-pyridin-2-ylmethyl)-1H-pyrimidine-2,4-dione	357.36	AIDS352909	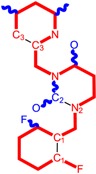	Fc1cccc(F)c1CN2/C=C\C(=O) N(C2=O)Cc3nc(C)cc(C)c3
					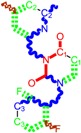	O=C1N(Cc(cc(C)c2)nc2C)C (N(/C=C1\)Cc(c(F)cc3)c(F)c3)=O
**25**	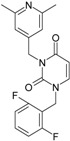	1-(2,6-Difluoro-benzyl)-3-(2,6-dimethyl-pyridin-4-ylmethyl)-1H-pyrimidine-2,4-dione	357.36	AIDS352910	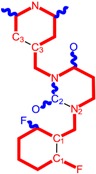	Fc1cccc(F)c1CN2/C=C\C(=O) N(C2=O)Cc3cc(C)nc(C)c3
					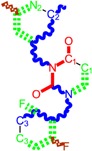	O=C1N(Cc(cc(C)n2)cc2C)C(N(/C=C1\) Cc(c(F)cc3)c(F)c3)=O
**26**	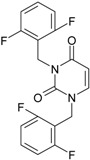	1,3-Bis-(2,6-difluoro-benzyl)-1H-pyrimidine-2,4-dione	364.30	AIDS352911	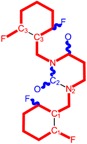	Fc1cccc(F)c1CN2/C=C\C(=O) N(C2=O)Cc3c(F)cccc3F
					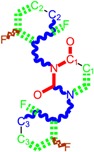	O=C1N(Cc(c(F)cc2)c(F)c2)C (N(/C=C1\)Cc(c(F)cc3)c(F)c3)=O
**27**	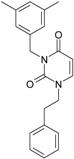	3-(3,5-Dimethyl-benzyl)-1-phenethyl-1H-pyrimidine-2,4-dione	334.42	AIDS352912	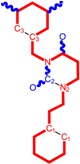	c1ccccc1CCN2/C=C\C(=O) N(C2=O)Cc3cc(C)cc(C)c3
					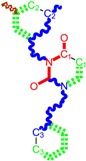	O=C1N(Cc(cc(C)c2)cc2C) C(N(/C=C1\)CCc(cccc3)c3)=O
**28**	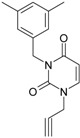	3-(3,5-Dimethyl-benzyl)-1-prop-2-ynyl-1H-pyrimidine-2,4-dione	268.32	AIDS352913	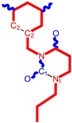	C#CCN1/C=C\C(=O) N(C1=O)Cc2cc(C)cc(C)c2
					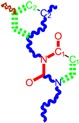	C#CCN1/C=C\C(=O) N(C1=O)Cc2cc(C)cc(C)c2
**29**	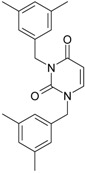	1,3-Bis-(3,5-dimethyl-benzyl)-1H-pyrimidine-2,4-dione	348.44	AIDS352914	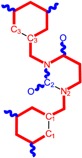	c1c(C)cc(C)cc1CN2/C=C\C(=O) N(C2=O)Cc3cc(C)cc(C)c3
					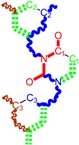	O=C1N(Cc(cc(C)c2)cc2C)C (N(/C=C1\)Cc(cc(cc3C)C)c3)=O
**30**	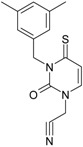	[3-(3,5-Dimethyl-benzyl)-2-oxo-4-thioxo-3,4-dihydro-2H-pyrimidin-1-yl]-acetonitrile	285.36	AIDS352915	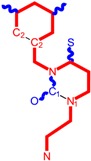	N#CCN1/C=C\C(=S)N (C1=O)Cc2cc(C)cc(C)c2
					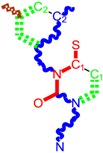	S=C1N(Cc(cc(C)c2)cc2C) C(N(/C=C1\)CC#N)=O
**31**	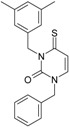	1-Benzyl-3-(3,5-dimethyl-benzyl)-4-thioxo-3,4-dihydro-1H-pyrimidin-2-one	336.45	AIDS352916	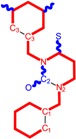	c1ccccc1CN2/C=C\C(=S) N(C2=O)Cc3cc(C)cc(C)c3
					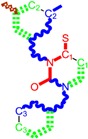	S=C1N(Cc(cc(C)c2)cc2C) C(N(/C=C1\)Cc(ccc3)cc3)=O
**32**	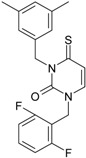	1-(2,6-Difluoro-benzyl)-3-(3,5-dimethyl-benzyl)-4-thioxo-3,4-dihydro-1H-pyrimidin-2-one	372.43	AIDS352917	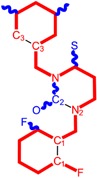	Fc1cccc(F)c1CN2/C=C\C(=S) N(C2=O)Cc3cc(C)cc(C)c3
					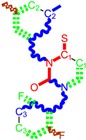	S=C1N(Cc(cc(C)c2)cc2C) C(N(/C=C1\)Cc(c(F)cc3)c(F)c3)=O

- The longest SMILES molecular chain (LoSMoC) is assumed to be the first stage in intermediary molecular defolding targeting the receptor. It is obtained by breaking one bond in every aromatic ring in the original molecule. The resulting “molecule” is displayed as a sort of 2D form for the original molecule, so casting a kind of “fractalic” chain; the maximum SMILES chain in LoSMoC is presumably responsible for best transport/transduction of ligand molecules through cellular (lipidic) walls. Afterwards they may be released with a modified structure due to their further ionization upon the interaction with cellular layers. Accordingly, another form of SMILES is generated and next to be considered, as follows;
- The Branching SMILES (BraS) represents the second conformation-phase of molecular defolding. It is obtained by ligand bonds’ breaking such that many “bays” are formed, yet with consistent “arms” linking the short molecular “skeleton” aiming to favor the binding with receptor in its pockets. Accordingly, the branching is not necessary in the same points of molecules through a series, yet an ”equilibrium” between maximum branching and stericity of branches accounts for the final BraS. For instance, a long branch adjacent to a short one will make an “anchor” not strong enough to bind the receptor pocket. Therefore, the “branching principle” requires having the equilibrated anchor-clefs by themselves. As such, the branching up to fourth order is performed for molecules in Table 1.

However, one should note the fact that most of the drugs are ionized once immersed in the biological body in accordance with the present two-steps of SMILES conformations. This happens because in each of these stages more nucleophilic compounds are considered due to the successive bond breaking and the loss of electronic pairs paralleling the defolding process from original to LoSMoC to BraS configurations.

These SMILES metabolic intermediates, posing nucleophilic active sides, are nevertheless confirmed at least for fused and non-fused diazines including those based on pyrimidines [100]. In this context, their antiviral and anti-HIV acting in special [101,102,103,104] and anti-inflammatory effects, in general, was already demonstrated [105,106,107].

Remarkably, the present SMILES analysis was also conceptually confirmed at least in two different ways, as follows:

➢ By the computational proof of the uncatalyzed racemization process where the openings and closures of the pyrimidinic nuclei happen just there where the above LoSMoc take places (Table 1). Therefore the concerned binding breaks go through a sort of SMILES transformations with a lower energy, following the principle of favoring the longest chain in molecular configuration. This is already a sort of structural variational principle in chemical bonding [108];
➢ By the recent QSARINS-Chem model for QSAR studies recognizing the role of SMILES canonical rules in correctly assessing the query and parsing the structure-activity algorithm (for the LoSMoC and BraS conformations, for instance) [109].

Worth noting is that the QSAR analysis performed by some of the present authors already revealed the general mechanism of action for the Genuine-LoSMoC-BraS related configuration for the actual pyrimidines’ anti-HIV ligands of Table 1 [97,98,99]. Yet, assessing of the highest propensity of anti-HIV binding for the individual molecule in the actual set of working compounds remains the aim of this work. The previous limitation is natural due to the statistical nature of the QSAR approach working with a pool of molecules while, for individual identification of the “most active” molecule in the sense of binding-and-conformation, the complementary analysis should be performed. A viable route in this regard is the docking approach by which also the amino acid residues’ presence assisting the bonding-conformation of a certain molecule may be predicted. This procedure is presented below with the discussion of the results in the forthcoming section.

## 3. Docking of Anti-HIV 1,3-Disubstituted Uracil Derivatives

### 3.1. Docking Algorithm

Molecular docking is used to predict the noncovalent binding of two molecules (usually a protein as the receptor molecule and a small ligand) and the affinity of binding. It starts with their unbound three-dimensional structures extracted from databases or obtained by computational methods. In our study we use, as a receptor molecule, the HIV-1 reverse transcriptase (HIV-1RT) and, as small ligands, a collection of 32 uracil derivatives with anti-HIV potential [97,98,99].

There are numerous three-dimensional structures of complexes made by the HIV-1RT with inhibitors in the Protein Data Bank (PDB) [110]. We have chosen in our study the high-resolution structure (1.8 Å) of the complex of HIV-1RT with (4-{[4-({4-[(E)-2-cyanoethenyl]-2,6-dimethylphenyl}amino)pyrimidin-2-yl]amino}benzonitrile (also called TMC278 or rilpivirine), a highly effective drug in treating wild-type and drug-resistant HIV-1 infections in clinical trials [111]. This structure of the HIV-1 RT heterodimer, in complex with the TMC278 drug, has the PDB code entry 2ZD1 and reflects an open-cleft conformation resembling those seen in other complexes of RT with nonnucleoside reverse transcriptase inhibitors (NNRTIs) [32,42,112]. The binding site of the drug is a hydrophobic tunnel connecting the NNRTI-binding pocket to the nucleic acid-binding cleft and is composed by residues belonging to both monomeric chains: L100, K101, K103, Y188, Y188, F227, W229, L234, H235, and P236 of the A chain (RT p66 subunit) and E138 of the B chain (RT p51 subunit) [111]. In our study we have considered, for the receptor structure, only the region containing the binding site of the TMC278 drug. The receptor has been prepared for molecular docking calculations by cleaning the heteroatoms and adding charges using the DockPrep facility under the UCSF Chimera package [113] UCSF Chimera package is also used for molecular docking outcome visualization and interpretation.

For molecular docking studies we used two online servers, PatchDock [114] and 1-Click Docking [115], based on distinct approaches for predicting the ligand-receptor complexes. PatchDock webserver performs structure prediction for both protein-protein and protein-small ligand complexes using a geometry-based molecular docking algorithm [114]. This algorithm is based on finding the transformations that produce a local good geometric shape complementarity by considering wide interface areas and small amounts of steric clashes, considering wide interface areas guarantees the inclusion of the local individualities of the docked molecules with complementary features. The transformations are classified using a scoring function that takes into account both the geometric fit and atomic desolvation energy and the redundant solutions are rejected by applying a root mean square deviation (RMSD) clustering. In our calculations we have used blind docking with default options, a clustering RMSD value of 4 Å, and “protein-small ligand” as complex type. FireDock webserver [116] has been used to refine the PatchDock predictions. It delivers the global energy of each enzyme-inhibitor complex predicted by PatchDock software.

The 1-Click Docking is an on-line server that predicts the binding orientation of a small ligand to a protein and gives a rough estimation of the binding affinity. It uses AutoDock Vina [115] with default parameters for docking calculations. AutoDock Vina is a docking algorithm based on a scoring function that approximates the standard chemical potentials of the molecular system. It is computed by combining empirical information concerning the conformational preferences of the receptor-ligand complexes and the experimental affinity measurements [115]. This server also gives information about the toxic potential of the ligand.

### 3.2. Docking Results

**Figure 4:**
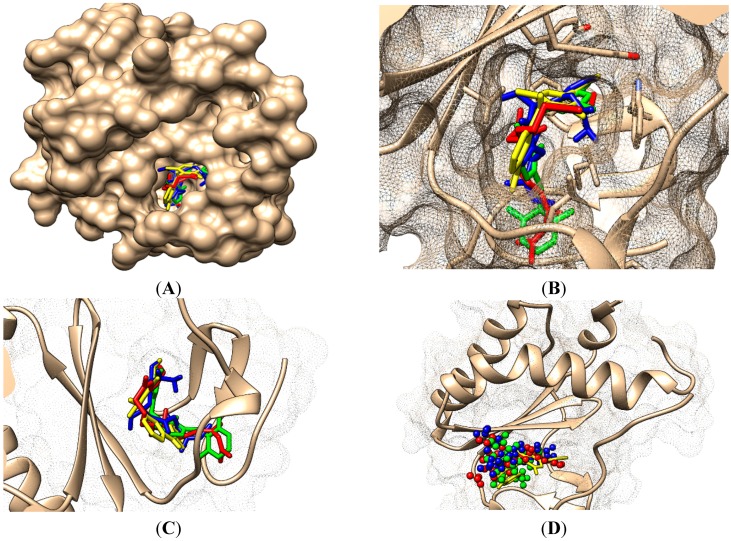
(**A**) The structure of HIV-1RT with rilpivirine drug bounded in a known place/*situs* to protein as our molecules; (**B**) more details on the docking of rilpivirine drug and the molecule no. 25 of Table 1; (**C**) still a different variant of 1-Click Docking results with molecule 25, yet with dashed surface and without adjacent amino acids; and (**D**) The PatchDock result of molecule no. 25, with the same color legend: the Genuine, BraS, and LoSMoC as green, red, and blue sticks, respectively.

Both the 1-Click Dock and PatchDock results for the binding of molecule 25 to HIV-1RT are presented in Figure 4: the protein is presented as backbone and continuous surface (brown), the drug rilpivirine is presented as yellow sticks, the Genuine molecule 25 as green sticks, the molecule 25 branched as red sticks and the molecule 25 as LoSMoC as blue sticks.

However, the results for all the molecular series in the Table 1 are displayed in the Table 2, Table 3 and Table 4 for the genuine, LoSMoc and BraS configurations, respectively.

**Table 2:** Results for the genuine molecules of Table 1 in terms of molecular surface and volume, docking algorithms as PatchDock and 1-Click Docking with the reported global energy and interface area—for the first approach and with the binding affinity and toxicity for the second one, respectively.

Molecule Genuine, No.	Molecular Area (Å^2^)	Molecular Volume (Å^3^)	PatchDock		1-Click Docking	
			Global Energy (kcal)	Interface Area (Å^2^)	Binding Affinity (kcal/mol)	Toxicity
1	318.3	358.2	−20.44	442.80	−9.30	Potentially toxic
2	333.7	386.2	−22.24	511.40	−9.40	Potentially toxic
3	326.8	363.1	−21.83	450.40	−8.30	Potentially toxic
4	342.4	388.7	−23.85	489.70	−8.70	Potentially toxic
5	342.4	390.2	−25.24	481.00	−9.40	Potentially toxic
6	331.6	387.0	−22.86	498.10	−9.60	Potentially toxic
7	351.9	396.1	−22.67	505.00	−9.70	Potentially toxic
8	345.3	391.0	−22.97	505.10	−9.10	Potentially toxic
9	356.1	419.9	−24.63	546.00	−9.20	Potentially toxic
10	318.2	345.7	−20.48	467.00	−5.80	Potentially toxic
11	369.7	417.4	−23.89	549.00	−8.20	Potentially toxic
12	398.3	462.5	−27.82	567.70	−7.40	Potentially toxic
13	351.1	395.9	−25.03	526.70	−7.20	Potentially toxic
14	353.0	393.1	−23.19	503.40	−6.50	Potentially toxic
15	392.1	438.1	−24.34	560.90	−7.70	Potentially toxic
16	349.9	393.7	−23.11	511.20	−10.4	Nontoxic
17	371.5	422.9	−25.89	546.00	−10.2	Nontoxic
18	422.3	489.4	−27.34	587.30	−11.3	Nontoxic
19	414.9	483.3	−26.99	587.80	−10.8	Nontoxic
20	444.0	515.1	−28.83	675.00	−10.7	Nontoxic
21	433.1	501.9	−26.78	648.00	−11.8	Nontoxic
22	354.3	399.4	−24.97	509.10	−10.00	Nontoxic
23	426.6	496.1	−23.31	561.70	−11.10	Nontoxic
24	424.4	488.1	−28.24	616.30	−8.00	Nontoxic
25	422.1	486.2	−24.82	616.30	−10.50	Nontoxic
26	380.9	438.0	−23.53	543.50	−10.40	Nontoxic
27	439.4	531.0	−26.85	657.80	−11.10	Nontoxic
28	363.9	406.1	−25.77	512.00	−9.50	Potentially toxic
29	427.9	556.1	−29.82	707.10	−11.50	Potentially toxic
30	354.4	399.9	−23.58	506.00	−9.20	Potentially toxic
31	427.7	493.1	−26.48	594.70	−11.00	Potentially toxic
32	427.7	497.8	−25.84	630.80	−11.3	Potentially toxic

**Table 3:** The same type of results as in Table 2, yet here for the LoSMoC configuration of molecules of Table 1.

Molecule Branched	Molecular Area (Å^2^)	Molecular Volume (Å^3^)	PatchDock		1-Click Docking	
			Global Energy (kcal)	Interface Area (Å^2^)	Binding Affinity (kcal/mol)	Toxicity
1	331.5	380.4	−22.09	473.00	−7.40	Potentially toxic
2	336.3	376.7	−20.69	488.80	−4.60	Potentially toxic
3	335.9	375.7	−23.66	492.60	−4.70	Potentially toxic
4	347.2	404.5	−25.32	519.20	−6.00	Potentially toxic
5	349.7	397.6	−23.10	505.20	−5.80	Potentially toxic
6	346.1	400.4	−22.69	489.00	−7.60	Potentially toxic
7	361.3	410.0	−24.06	534.60	−7.40	Potentially toxic
8	356.3	406.3	−24.02	509.01	−7.60	Potentially toxic
9	365.2	431.1	−26.85	536.70	−8.10	Potentially toxic
10	327.4	359.4	−21.72	454.00	−7.00	Potentially toxic
11	380.0	424.9	−22.46	529.70	−5.20	Potentially toxic
12	406.1	474.6	−24.31	591.40	−5.50	Potentially toxic
13	384.7	409.6	−22.06	506.80	−7.90	Potentially toxic
14	387.3	413.5	−22.16	510.20	–	Potentially toxic
15	400.3	446.4	−28.20	605.70	–	Potentially toxic
16	370.1	412.6	−22.53	502.10	−8.20	Potentially toxic
17	397.1	449.0	−27.21	580.10	−8.30	Potentially toxic
18	442.5	509.4	−26.17	655.10	−6.10	Potentially toxic
19	440.3	501.3	−28.46	633.20	−8.00	Potentially toxic
20	462.3	539.0	−26.17	644.20	−8.60	Potentially toxic
21	448.7	515.7	−23.04	620.80	−6.30	Potentially toxic
22	372.3	414.4	−23.59	515.10	−8.00	Potentially toxic
23	440.4	509.4	−21.71	640.60	−8.30	Potentially toxic
24	438.2	501.1	−24.35	635.40	−8.20	Potentially toxic
25	439.6	501.1	−24.44	647.80	−7.80	Potentially toxic
26	397.7	451.8	−23.62	561.50	−8.00	Potentially toxic
27	461.2	536.0	−26.39	538.50	−8.20	Potentially toxic
28	368.2	413.0	−22.06	522.40	−7.70	Potentially toxic
29	486.9	569.1	−25.86	582.70	−8.20	Potentially toxic
30	362.7	410.5	−24.05	544.80	−7.80	Potentially toxic
31	442.9	512.5	−25.96	638.20	−8.20	Potentially toxic
32	445.6	515.6	−27.96	648.00	−8.30	Potentially toxic

Nevertheless, the results of Table 2, Table 3 and Table 4 are to be analyzed in light of the conformational-binding variational approach of the Genuine-LoSMoC-BraS transformations as in the sequel presented and discussed.

**Table 4:** The same type of results as in Table 2 and Table 3, yet here for the BraS configuration of molecules of Table 1.

Molecule Branched	Molecular Area (Å^2^)	Molecular Volume (Å^3^)	PatchDock		1-Click Docking (AutoDock Vina)	
			Global Energy (kcal)	Interface Area (Å^2^)	Binding Affinity (kcal/mol)	Toxicity
1	326.5	369.3	−21.18	485.20	−7.60	Potentially toxic
2	336.5	376.9	−23.16	493.90	−8.50	Potentially toxic
3	336.0	375.8	−25.23	460.00	−4.80	Potentially toxic
4	347.9	404.4	−24.99	501.60	−7.90	Potentially toxic
5	349.7	397.6	−23.10	505.20	−8.00	Potentially toxic
6	346.3	400.3	−23.43	514.70	−7.80	Potentially toxic
7	361.2	409.9	−23.06	547.80	−8.50	Potentially toxic
8	356.2	406.2	−24.23	534.00	−7.50	Potentially toxic
9	365.2	431.7	−27.04	549.00	−8.20	Potentially toxic
10	327.4	359.5	−23.38	631.10	−7.40	Potentially toxic
11	380.0	424.9	−22.46	529.70	−4.40	Potentially toxic
12	406.6	474.7	−26.03	582.90	−5.40	Potentially toxic
13	384.8	409.6	−21.70	478.10	–	Potentially toxic
14	387.3	413.4	−21.12	505.00	−8.10	Potentially toxic
15	400.4	446.5	−26.23	551.60	−5.90	Potentially toxic
16	370.7	413.1	−23.37	529.00	−4.30	Potentially toxic
17	397.5	451.6	−24.88	559.50	–	Potentially toxic
18	442.5	509.1	−27.12	669.40	−5.10	Potentially toxic
19	440.7	501.7	−27.25	611.10	−4.90	Potentially toxic
20	462.2	538.5	−26.01	611.90	−4.60	Potentially toxic
21	448.7	517.7	−21.27	652.50	−5.60	Potentially toxic
22	372.5	414.5	−23.57	533.30	−8.60	Potentially toxic
23	440.5	509.2	−23.94	592.30	−5.10	Potentially toxic
24	438.0	500.4	−23.90	626.80	−5.00	Potentially toxic
25	439.3	501.2	−23.70	596.90	−9.30	Potentially toxic
26	399.5	454.1	−24.27	576.00	−10.10	Potentially toxic
27	461.4	536.5	−27.21	677.00	−5.60	Potentially toxic
28	368.4	412.9	−22.02	540.60	−8.20	Potentially toxic
29	486.7	568.8	−22.23	681.00	−8.80	Potentially toxic
30	362.9	410.4	−24.80	518.90	−4.20	Potentially toxic
31	443.0	512.8	−25.04	655.50	−5.00	Potentially toxic
32	445.7	515.7	−23.38	631.10	–	Potentially toxic

## 4. Discussion: Variational Binding-Conformational Analysis

### 4.1. The Double Variational Output

The present variational approach combines the binding with conformation information in establishing the best molecular structure, out of compounds of Table 1 that best fits in the HIV-1-RT pocket towards inhibiting its activity. The actual variational principle states in the first stage that:

➢ Minimum (in negative, so favoring the binding) energy (either as affinity and/or global) associated with a toxically potent molecule highly recommends that structure for the binding purpose, according with the performed docking algorithm.

Then, one implements the second variational stage by:

➢ Binding variational procedure across the various conformations of a compound, such as Genuine, LoSMoC and BraS, towards further providing binding-conformational best regarded molecule(s) for the aimed anti-viral activity.

Practically, our “best anti-HIV molecule” results in that structure with toxic potential found at the intersection between minimum (in negative, so favoring the binding) affinity, global energy, and all tested configurations (Genuine, LoSMoC, and BraS).

Therefore, implementing a kind of triple criteria of selection (*i.e.*, the two above variational steps plus their overlapping results), should assure the common binding- and configurational-variational behavior of the selected molecule; this way “ably fighting” the natural shielding versatility of the HIV-RT pocket in these regards.

By analyzing the concrete results of Table 2, Table 3 and Table 4 for the molecules of Table 1, one arrives to the following hierarchies:

➢ Variational binding affinity procedure selects the following toxically-potent molecules:
  Genuine: 18, 21, 29;
  LoSMoC: 16, 17, 20, 23, 24, 27, 29, 31, 32;
  BraS: 25, 26, 29;

➢ Variational global energy procedure selects the following toxically-potent molecules:
  Genuine: 20, 24, 29;
  LoSMoC: 15, 17, 19, 27, 32;
  BraS: 18, 19, 27;

➢ Now we are in position to identify the “first intersection” regarding the recorded double outputs per configuration (Genuine, LoSMoC, and BraS) while passing from binding affinity to global energy minimums:
  Genuine: 29;
  LoSMoC: 17, 27, 32;

Therefore, excluding the BraS contribution as not so versatile, according to this inter-variational criteria, while selecting the single molecule no. 29 in its Genuine configuration as the potential candidate.

➢ Performing the “second intersection” regarding the multiple outputs inter-configurations (among Genuine, LoSMoC, and BraS) while maintaining either binding affinity or global energy framework:
  18: Genuine & BraS;
  19, 27: LoSMoC & BraS;
  20, 24: Genuine & LoSMoC;
  29: Genuine, BraS, LoSMoC;

Therefore, selecting a single molecule for Genuine and BraS, namely molecule no. 18, yet reconfirming molecule no. 29 as the common output for all inter-configuration variations.

**Figure 5:**
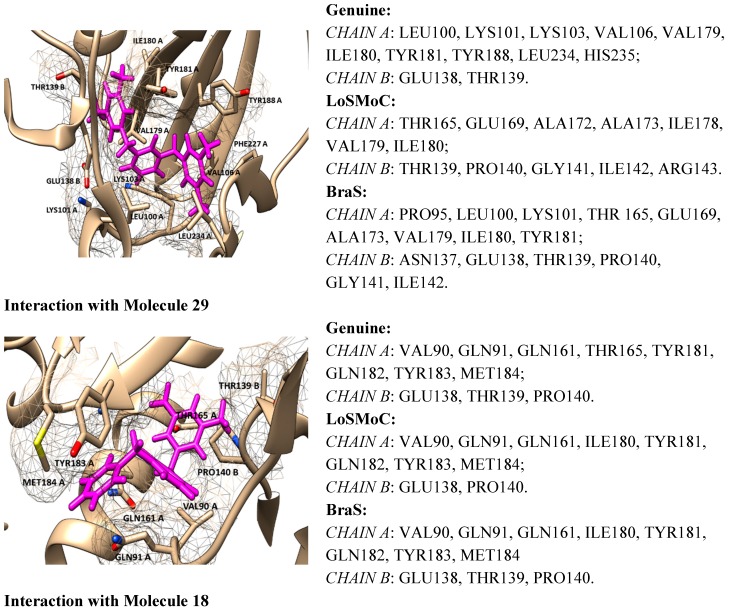

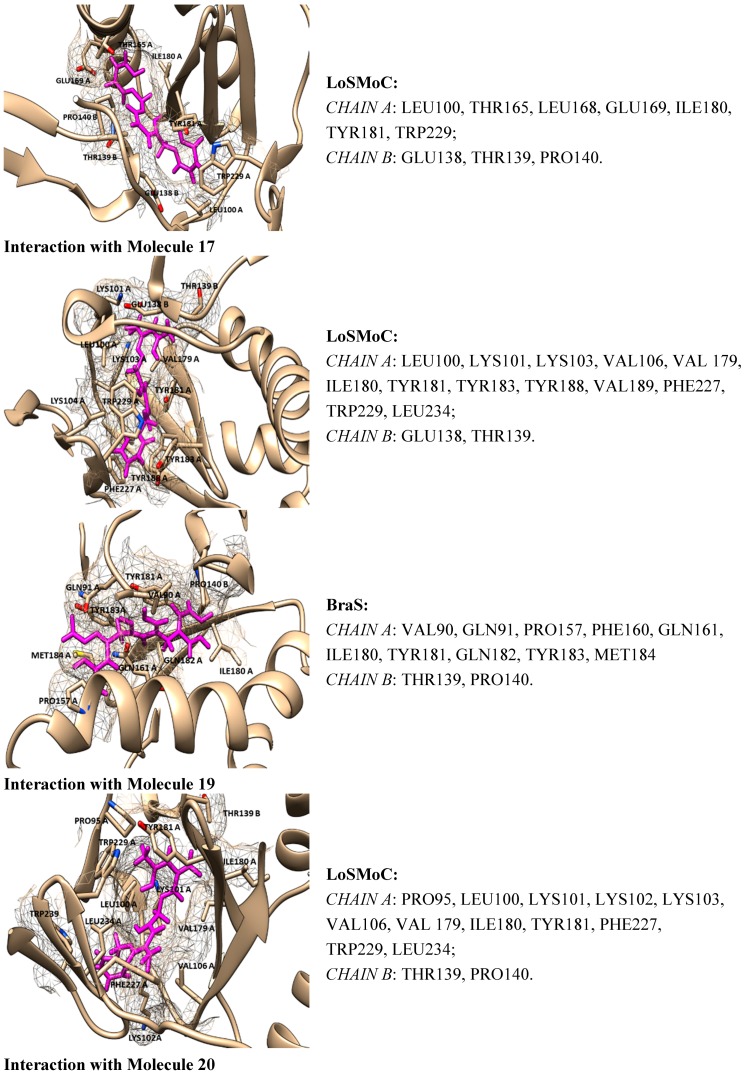

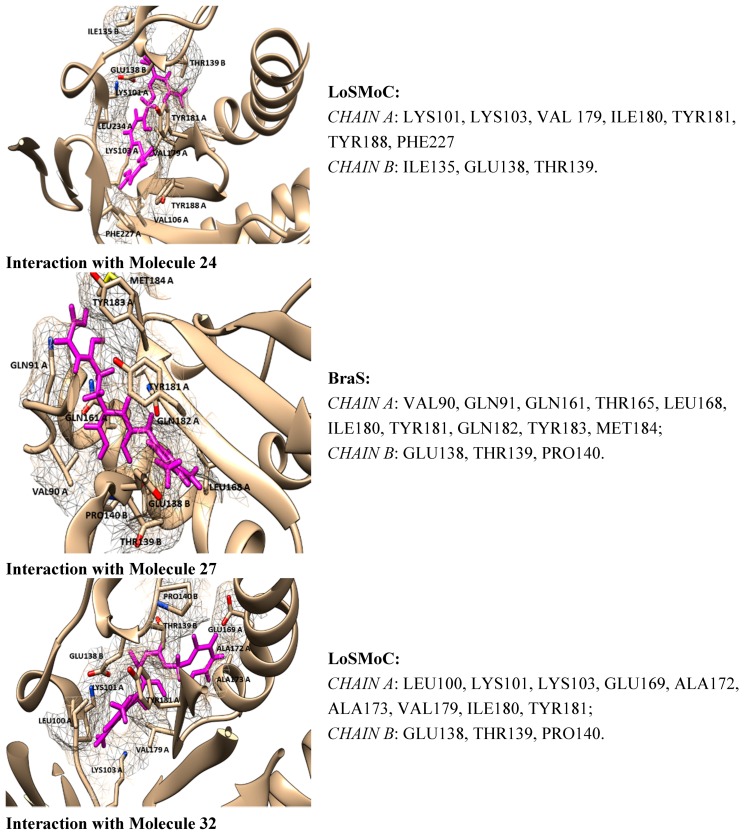
ContPro [117] results for interacting residues with selected molecules at 3.5 Å by the present Genuine-LoSMoC-BraS double variational procedure, in their relevant configuration(s), see text for details.

As a result of per molecules per configurations, the selected molecules in an actually-proposed hierarchy of their anti-HIV potency by variational binding-conformational docking analysis looks like:

29: Genuine, LoSMoC, & BraS;
18: Genuine & BraS;
17: LoSMoC;
19: LoSMoC & BraS;
20: Genuine & LoSMoC;
24: Genuine & LoSMoC;
27: LoSMoC & BraS;
32: LoSMoC;

Overall the output is that molecule no. 29 of Table 1 appears as our best selection by the actual doublevariational procedure (binding and conformational analysis). However, the interactions with amino acids found closer than 3.5 Å are shown in Figure 5 for the above double variationally-selected molecules.

### 4.2. Discussing the Interaction with Amino Acids

The binding results of selected molecules by double binning-conformational variational algorithm and by their anti-HIV interaction are illustrated by the Docking package in Figure 5 with available amino acids by their certain (Genuine, LOSMoC and BraS) configurations. They are to be commented on next, in the general context of anti-HIV pyrimidine available knowledge.

Many compounds with anti-HIV activity from the NNRTI inhibitor class include, in their molecule, two or three aromatic rings which can form hydrophobic (e.g., π–π) interactions with NNBP. In the case of the pyrimidine-derivative NNRTI inhibitors, one of the aromatic nuclei is the pyrimidine (in many cases being the uracil) and the other core is the benzyl group, a common and important ingredient in maintaining the anti-HIV activity of the molecule.

Benzyl or dimethyl-benzyl core also can be found in the structure of some pyrimidine derivatives INI inhibitors of HIV-1RT or dual inhibitors INI + NNRTI. In these cases the bond is in a C-6 or C-2 position of the pyrimidine core, so the diketo acid (DKA) structure (required to bind two divalent metal ions Mg^2+^ or Mn^2+^) is linked in N1/N3 position in case of INI inhibitors. In case of dual inhibitors, the chelating triad of Mg^2+^ can be included in pyrimidine core (used an uracil core, [C/N_positionX_]): [C_p2_]O-[N_p3_]H(or OH)-[C_p4_]O, where the groups of benzyl or dimethyl-benzyl are bounded in N-1 or C-6 positions [20,21,22,118]. However, for the actual majority of pyrimidine derivatives NNRTIs inhibitors with an excellent anti-HIV-1 activity of HIV-1 (as HEPT, DABO, DAPY) the following findings are specific [21,28,39,40,48,50,68,69,70,71,72,73,74,75,76,77,78,79,80,81,82,83,84,85,86,89,90,119,120]:

➢ The position of the molecule is in the hydrophobic region of the NNRTI binding site/hydrophobic interactions (by π–π, π-CH, van der Waals contacts) having as two major substituents of the pyrimidine core the residues Tyr181, Tyr188, Phe227, Trp229, His235, Pro238 and/or Val106;
➢ The –CH_2_– linker of benzyl group or methyl group bound to the benzene ring is positioned closely to Glu138 from the p51 domain of RT, while the pyrimidine core is positioned in the area between Leu100 and Val179;
➢ The formation of one or more H-bonds with Lys101 (and/or Lys103) where there are possible;
➢ The Ar-H interactions with Leu234 are often observed.

Worth noting is that, in many known cases, the –CH_2_– linker is replaced with others like –S–, –NH–, –O–; or, the hydrogen atoms from –CH_2_– linker is replaced with –OH and/or –alkyl groups. These changes lead to an increase in anti-HIV activity for many derivatives (such as DABO and DAPY inhibitors) due to a better binding to NNIBP. Working examples are –NH–, –O–, and –CH(OH)– groups that can be involved in H-bonding with the Lys101 in DAPY-type inhibitors [37,50,69,70,71,72]. In some cases, the former types of substitutions will lead to a more hydrophobic space occupied in NNIBP.

Benzyl groups, with or without –CH_2_– linker modified, are usually involved in hydrophobic interactions, π–π, and/or arenas-H with one or more residues Tyr181, Tyr188, Phe227, Trp229 from NNIBP [21,28,39,40,48,50,68,69,70,71,72,73,74,75,76,77,78,79,80,81,82,83,84,85,86,89,90,119,120]. The number and type of these interactions differ more or less from one compound to another, no matter the NNRTI family they belong to. This behavior is due to the cumulative effects of other existing substituents in the molecule and, in the case of a chiral compound [69], the differences between enantiomers can be significant. Accordingly, the orientation of the “U” shape for one of the enantiomers is downward, which can be nearly opposite to that of the usual “U” shape, and can lead to a weak inhibition against HIV-1 RT [69].

In general, when –CH_2_– linker is extended it will lead to a weak inhibition of HIV-1 RT, e.g., for DABO and HEPT inhibitors, excepting some derivatives where a deeper penetration into the pocket is observed [39,40,59,60,61,62,63,64,65,66,77,78,79,80,81,82,83,84,85,86,92,93,94,95]. However, the compounds from the presently-studied series, and in all of their three forms (Genuine, LoSMoC, and Branch), bind in non-classical “U” mode, since the “U” is a bit twisted and it has two or more interactions specified for pyrimidine NNRTI derivatives.

On the other hand, for the variationally-selected compounds 20 and 24 the presence of a majority of amino acid residues it was observed at a distance of less than 3.5 Å:

➢ For compound 20/LoSMoC: Leu100, Lys101, Lys103, Val106, Val179, Tyr181, Phe227, Trp229, Leu234;
➢ For compound 20/Genuine: Lys101, Lys103, Val106, Val179, Tyr181, Tyr188, Phe227, Trp229, Leu234, His235, Pro236;
➢ For compound 24/LoSMoC: Lys101, Lys103, Val179, Tyr181, Tyr188, Phe227, Glu138B;
➢ For compound 24/Genuine: Leu100, Lys101, Lys103, Val106, Val179, Tyr181, Tyr188, Phe227, Trp229, Leu234, Glu138B.

with the first two amino acid residues groups being deep in the hydrophobic pocket.

According with the current variational results, the hydrophobic interaction of the molecule with Tyr181 can be observed in most of the cases, not taking into account the compound form (as Genuine, LoSMoC, or Branch). This behavior underlines once again that for a better inhibition of RT HIV-1 the 1,3-disubstituted uracil derivatives widely features the interaction with Tyr181, similar with the results produced by Maruyama *et al.* [28,48,87,88,89,90,91]. This way, the importance of this interaction provides the explanation for the dramatic decrease in the inhibitory activity of these compounds against mutant Y181C. In the case of actual compound 18 (BBF29), *i.e.*, the (Maruyama) leader from which the development of AmBBU (as derivatives with poor activity against mutant Y181C) was started, all its three forms (Genuine, LoSMoC, Branch) interact with Tyr181 and Glu138 from the p51 domain. Moreover, the docking studies of the compound 18/AmBBU derivative show that this inhibitor interacts with the pool of amino acid residues Leu100, Val106, Tyr181, and Trp229, suggesting its inhibitory effect on HIV-1 RT (wt) [28].

The arene-H interactions such as:

➢ Leu100 (H) with the central aromatic ring (2-pyrimidine) of AmBBU;
➢ Val106 (H) with 1-benzyl of AmBBU;
➢ Tyr181 (arene) with hydrogen (3-methyl) at 3-(3,5-dimethylbenzyl) of AmBBU;
➢ Trp229 (arene) with 4ʹ-hydrogen of 3-(3,5-dimethylbenzyl) of AmBBU.

were equally observed [28], with the conclusion that the interaction with the Tyr181 residue is essential for docking of 6-substituted-1-benzyl-3-(3,5-dimethylbenzyl)uracils. The precise role of the 6-amino substitution in the binding to the allosteric pocket could not be identified [28].

For a series of 1-benzyl-3-(3,5-dimethylbenzyl) uracil and related compounds, which do not form an H-bond (with Lys101, usual necessary), a remaining affinity with RT was observed indicating that the H-bond is not essential for docking of such small molecules [48]. For the “brother” of AmBBU, the compound XXIV, the docking studies show that [91]:

➢ The 6-amino group forms a H-bond with Lys 101 (due to water solubility of the 4-aminobenzyl group of XXIV);
➢ The 3,5-dimethylbenzyl moiety enhanced the π–π stacking of the benzene rings of the Tyr181 and Tyr188 residues;
➢ The CH–π interactions are manifested between the methyl group of the 3,5-dimethylbenzyl moiety and Trp229 residue, or between the benzene rings of the 3,5-dimethylbenzyl moiety and Leu234 residue.

Generally, it is apparent that the position near the Glu138B of the –CH_2_– linker of the benzyl group of the methyl groups aids the binding of the benzene ring. This is observed in the majority of the actually-studied compounds in all three molecular conformations, namely:

➢ LoSMoC favoring cellular penetration;
➢ Branch favoring “binding” to the active site;
➢ and Genuine towards restoring the original molecule, the actually inhibition.

This advanced hierarchical mechanism suggests that these 1,3-disubstituted uracil derivatives use Glu138B as a compass or a guiding point towards a better placement of the molecule in the active site of RT HIV-1, yielding with the inhibition of its activity.

This mechanism is finally discussed for the overall output of this variational study, compound 29. While containing in its structure the 3,5-dimethyl-benzyl group bound in both N1 and N3 positions of the pyrimidine core, it appears to be the most potent molecule by the actual variational anti-HIV Genuine-to-LoSMoC-to-Branch principle:

➢ By the LoSMoC configuration it features the specific interactions of pyrimidine NNRTI derivatives only with Val179, so predicting the future position of the pyrimidine core;
➢ On its Branch form the presence of new amino acid residues specific to NNRTI-pyrimidines are observed: Glu138B, Lys101, Val179 and Leu100; the latter two are forming the future space where pyrimidine core will be set in, with the specific placement of pyrimidine substituent in the hydrophobic area being delimited, among others in its entry of Figure 5, by Tyr181;
➢ In the Genuine conformation the compound 29 has the correct “U” shape of molecule with a little twist, so keeping in its proximity the same amino acid residues as in the previous Branch form, along the additional ones: Lys103, Ile180, Val106, Tyr188, Leu234, and His235.

The Genuine molecule 29 finally acts as:

➢ Restoring the pyrimidine core in the area between Val179 and Leu100;
➢ Having the –CH_2_– linker of the benzyl group placed closely to Glu138B;
➢ Having the methyl groups from 3,5-dimethyl-benzyl and the benzene ring deep positioned in the hydrophobic pocket, closed to Val106, Tyr188, Leu234, and His235 (forming hydrophobic interactions with them);
➢ Having the other 3,5-dimethyl-benzyl substituent placed close to Tyr 181 and Ile180.

## 5. Conclusions

Pyrimidine derivatives occupy a favored position among the compounds investigated/approved for anti-HIV activity.

The presence of pyrimidine core is obligatory in NRTIs and NtRTI inhibitors, and they are increasingly used in the design of NNRTIs, INIs, NcRTIs, PIs, CRIs, and dual inhibitors. As microbicides they are often substituted in various positions with (3,5-dimethyl)-benzyl, the more important HEPT derivatives become. They provide front-runner compounds and inspire researchers to develop other pyrimidine families’ derivatives with anti-HIV activity, e.g., the DABO, DAPY pyrimidindione derivatives. They are at the forefront in the treatment of HIV, with pharmacotherapy and antiviral therapy. However, many side effects have arisen, and there is a continual battle with drug toxicity paralleling HIV mutations that become more resistant. In the pharmacotherapy of HIV infection there is a common practice to use the combination therapy of three or more antiretrovirals (ARVs) dealing with different targets. This therapy was introduced in the mid-1990s, being called highly active antiretroviral therapy (HAART) and over the years it has led to a decreased morbidity and mortality among HIV-infected patients [25,26,27,29,121,122,123]. Although more than 25 years have passed since the first approved ARV for treating HIV infection, a treatment with constant high rate of healing of any HIV subtypes or mutations it hasn’t been found yet, nor an inexpensive administrated treatment, accessible to large social classes. In addition, finding a treatment based on a near future vaccine or on effective microbicides to be widely used as a method of HIV prevention is a humankind desiderata.

**Figure 6:**
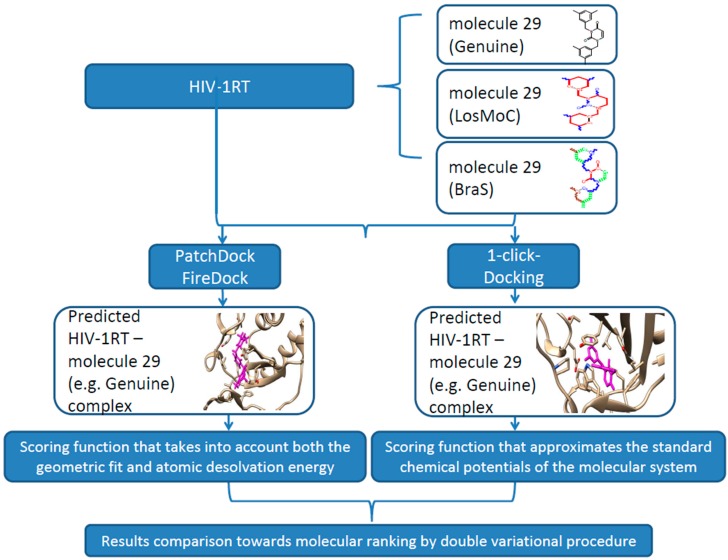
Flow diagram summary outlining the computational protocol towards double variational procedure in the relevant configuration(s) for the present outlined molecule 29; See text for details.

In this context, the present work contributes to currently-available studies in assessing the anti-HIV activity by small, flexible, and easy to synthesize molecules—pyrimidine derivatives in this case. Moreover, the actual approach advances the double-variational procedure, combining the binding (affinity and total energy) with molecular conformation (by Genunine and LoSMoC and BraS SMILES forms) by available docking protocols for a representative series of 32 molecules with earlier-studied anti-HIV activities (resumed in Figure 6).

Nevertheless, the molecule identified as “most potent” by this double-variational docking-SMILES analysis, was molecule 29 across compounds of Table 1 and shows, at 3.5 Å, interaction with amino-acids of the HIV RT pocket significant similarities with previously-assigned potent anti-HIV molecules in this regard, namely the molecule no. 18 of Table 1. They are in proximity of the residues Tyr181, Glu138, and Thr139 for Genuine and Ile180, Tyr181, Glu138, and Pro 140 for BraS–SMILES forms, respectively, while molecule 29 complements the earlier analysis also by its LoSMoc conformational potent presence, with a triggering role in the present anti-HIV docking mechanism study, worthy to be further focused for research and testing.
